# Does the Possibility of Using Donor Human Milk Limit the Pursuit to Feed Neonates Their Own Mother’s Milk? The Impact of a Newly Opened Human Milk Bank on Feeding Practices in a Neonatal Intensive Care Unit, North-East Poland

**DOI:** 10.3390/nu18050830

**Published:** 2026-03-04

**Authors:** Monika Kamianowska, Barbara Bebko, Agata Ostasz, Joanna Sieńko, Aleksander Kamianowski

**Affiliations:** Department of Neonatology and Neonatal Intensive Care, Medical University of Bialystok, M. Sklodowskiej-Curie 24A, 15-174 Bialystok, Poland

**Keywords:** human milk, donor human milk, human milk bank, lactation, neonate, feeding

## Abstract

**Background**: Human milk is considered an ideal diet for neonates, and every effort should be made to promote breastfeeding. Donor human milk (DHM) remains the best alternative for neonates when their mother’s own milk (MOM) is not available. We tried to determine whether having easy access to DHM from a Human Milk Bank (HMB) would reduce the pursuit to feed neonates MOM. **Methods**: A retrospective study was conducted on data from neonates consecutively admitted to the Neonatal Intensive and Intermediate Care Units of the Department of Neonatology of the Medical University of Bialystok between 1 January 2022 and 31 March 2025. The study period covered 2 years before the opening of the HMB and 1 year of its operation. No specific changes in feeding practices occurred simultaneously during the HMB’s first year of operation. **Results**: In the first year of operation of the HMB, we observed an increase in the percentage of neonates who (1) received mother’s own colostrum (71.88% vs. 52.28% (2023) and 52.05% (2022); *p* < 0.001), (2) were fed human milk during hospitalization (24.38% vs. 3.57% (2023) and 4.09% (2022); *p* < 0.001) and (3) were fed MOM at discharge (43.86% vs. 56.25%, *p* = 0.024). In total, 53.06% of neonates who received DHM were fed MOM at discharge. **Conclusions**: The possibility of using milk from the HMB did not limit the desire to feed neonates MOM but intensified it. Neonates were more likely to be fed MOM during the first feeding, throughout their hospitalization, and at discharge. It shows the strong potential of HMBs in improving feeding practices in Neonatal Intensive and Intermediate Care Units.

## 1. Introduction

Human milk is considered an ideal diet for neonates that ensures their survival and health. Due to its rich composition, human milk fulfills the nutritional needs of neonates, supporting optimal growth and development [1]. According to World Health Organization (WHO) and United Nations Children’s Fund (UNICEF) recommendations, children should be exclusively breastfed for the first 6 months of life, and breastfeeding should be initiated within the first hour of birth [2,3]. However, contrary to these recommendations, many children under 6 months are not exclusively breastfed [2].

While every effort should be made to promote and support breastfeeding, donor milk remains the best alternative for neonates if their mother’s own milk is unavailable. It allows for the early initiation of enteral nutrition while avoiding the risks associated with formula [4]. According to WHO, preterm or low birthweight neonates, in the absence of mother’s own milk and when possible, should be fed with donor human milk obtained from a human milk bank [5]. Donor human milk is donated by an infant’s mother (honorary donor) to a human milk bank, when its quantity exceeds demand [6]. Then it is tested, safely stored, and delivered to an infant, based on clinical necessity [7]. However, the use of donor human milk should be understood as a temporary bridge leading to feeding fully with the mother’s own milk. It should not undermine breastfeeding but support it [8].

According to the European Milk Bank Association, currently (18 January 2026), in Europe, there are 282 active human milk banks. In Poland there are 16 such facilities, including the Human Milk Bank of the University Clinical Hospital of Bialystok, which was established on 12 January 2024 [9]. The opening of the Human Milk Bank in Bialystok was extremely important from the perspective of this specific region of Poland. Since the opening of the first polish Human Milk Bank in Warsaw on 28 March 2012, no such facility had operated in north-eastern Poland. The opening of the Human Milk Bank in Bialystok means that the region of north-eastern Poland is no longer a “blank spot” on the map of neonatal care [10].

However, as already mentioned, the desire to expand the activities of human milk banks should accompany the pursuit to support breastfeeding. The aim of the study was to determine whether having easy access to donor human milk from the Human Milk Bank would reduce the pursuit to feed neonates their own mothers’ own milk.

## 2. Material and Methods

### 2.1. Early Lactation Stimulation Program

For many years, we have been conducting intensive lactation monitoring at the Department of Neonatology and Neonatal Intensive Care Unit of the Medical University of Bialystok, based on carefully developed procedures. We were satisfied with the quality of feeding of the hospitalized children. However, this was a subjective assessment. In the third quarter of 2021, we conducted an objective analysis of the quality of neonatal nutrition at the Department (based on medical records), which showed a significant need to modify the procedures (in the 4th quarter of 2021, only 12% of neonates hospitalized in the Neonatal Intensive and Intermediate Care Units of the Department of Neonatology received colostrum in the first feeding). In January 2022, we introduced the “Early Lactation Stimulation Program” covering not only our Department but also the Department of Perinatology with the Delivery Room. We paid particular attention to collaboration between the Departments, education, and documenting lactation activities to monitor the effectiveness of the implemented program. We conducted quarterly analyses and introduced modifications. The data analyses showed that the quality of nutrition at our Department was improving quarter after quarter, but there were still situations in which we were unable to obtain colostrum—when the mother was unwell, taking medication, refusing early lactation stimulation, refusing to feed the child with her own milk, or was absent (a child was admitted from another hospital). Therefore, the analysis revealed a significant need for donor human milk from the Human Milk Bank, which was opened at the University Clinical Hospital of Bialystok in January 2024. Our first donor was qualified in March 2024.

The criteria for becoming a recipient of milk from the Human Milk Bank were consistent with the Polish health policy program of comprehensive family support “For Life” for the years 2022–2026, whose goals included, among others, the nutrition of neonates and infants with human milk. The following criteria were used: (1) gestational age of 23 0/7-36 6/7 weeks, (2) chronological age of ≤28 days, (3) feeding via gastric tube (all feedings within 24 h) [11]. The nutrition was still analyzed as a part of the “Early Lactation Stimulation Program”, because there was a concern that the use of donor human milk from the Human Milk Bank would negatively impact the current implementation of the program, and our efforts to feed children with their mothers’ own milk would decrease.

### 2.2. Subjects and Data Collection

A retrospective descriptive and analytical study was conducted on data from all neonates consecutively admitted to the Neonatal Intensive and Intermediate Care Units of the Department of Neonatology and Neonatal Intensive Care Unit of the Medical University of Bialystok during the study period. The study center is located in the main tertiary referral hospital in north-eastern Poland. It is a representative of medical facilities offering neonatal care in this region, where the highest number of premature babies is born. In other hospitals, premature births are rare, and these facilities rarely need donor human milk. During the study period, our Department needed 85,500 mL of donor human milk. Other hospitals in the region required only 5700 mL.

The study period covered 3 time periods: (1) year 2022: from 1 January 2022 to 31 December 2022, (2) year 2023: from 1 January 2023 to 31 December 2022, (3) the first year of actual activity of the Human Milk Bank: from 1 March 2024 to 31 March 2025. The neonates born between 1 January 2024 and 29 February 2024 were excluded from the study to enable analysis in annual cycles, as it was the start-up period of the HMB.

During the study period, 527 neonates were admitted to the Units. However, the number of neonates subjected to further analysis was reduced by 28 (5.31%), because we excluded neonates who received exclusively total parenteral nutrition due to medical reasons.

The analysis was based on data collected as a part of the Early Lactation Stimulation Program. A structured questionnaire was used to collect data correctly. The following variables were included in the questionnaire: gestational age, birth weight, mode of delivery, place of birth (inborn, outborn), type of pregnancy (single, multiple), gravidity and parity. We paid special attention to collecting information about the methods of first feeding, feeding during hospitalization, and at discharge from the Department. To ensure patients’ privacy, only encoded data were entered into the database.

### 2.3. Data Management Plan

Firstly, we generated an enrollment code for all neonates included in the study. Then, they were entered into the database using the enrollment code. Our questionnaire consisted of four parts. The first part was used to enter patient data collected from the medical records. The second part included data regarding the methods of the first feeding, feeding during hospitalization, and at discharge from the Department. The third part assessed a neonate’s suitability for the study. Firstly, we checked for missing data. In addition, when a neonate received exclusively total parenteral nutrition due to medical reasons, it was excluded from the study. In the fourth part, we assessed the reasons for not receiving milk from DHM. This part only concerned 2024.

### 2.4. Operational Definitions

In terms of feeding methods, the following terms were used: (1) MOC—mother’s own colostrum, (2) MOM—mother’s own milk, (3) DHM—donor human milk, (4) FM—formula, (5) HM—human milk (including MOM, MOC, and DHM).

### 2.5. Sample Size and Missing Data Points

Because of the nature of the study, sample size calculations were not performed. We used all available data from the medical records. We decided to use a “complete case analysis” and exclude all cases with missing data. However, the medical records of all neonates admitted to the Neonatal Intensive and Intermediate Care Units of the Department during the study period were complete.

### 2.6. Data Quality Control

Three days of training were mandatory for all data collectors (four researchers) and covered the following aspects: data collection, handling and storage. During the training, it was emphasized that administration of even 1 mL of FM disqualified a neonate from the group of children fed exclusively MOM. The process of data collection was monitored by the principal investigator. After completing, we checked the questionnaires for data consistency and completeness. All corrections were performed before data analysis.

### 2.7. The Assessment of First Feeding

The neonates were divided into 3 categories, based on the type of milk they received: (1) MOC, (2) DHM, (3) FM. In 2022 and 2023, the neonates could receive only MOC or FM. In the first year of operation of the HMB, all three options were possible.

### 2.8. The Assessment of Feeding During Hospitalization

Initially the neonates were divided into 3 categories, based on the type of milk they received: (1) MOM, (2) mixed: MOM + FM, (3) FM. Then, when we analyzed data from the first year of operation of the HMB, three additional groups were distinguished: (4) MOM + DHM, (5) MOM + DHM + FM, (6) FM + DHM.

The methods of feeding were assessed also in individual gestational age groups: (1) extremely preterm: 23 0/7–31 6/7 weeks of gestation, (2) moderately preterm: 32 0/7–33 6/7 weeks of gestation, (3) late preterm: 34 0/7–36 6/7 weeks of gestation, (4) term: 37 0/7–42 6/7 weeks of gestation.

Then, we wanted to check what percentage of neonates required/did not require DHM and what percentage of them finally received it. The reasons for not receiving milk from DHM were also analyzed.

### 2.9. The Assessment of Feeding at Discharge from the Department

The neonates were divided into 3 categories, based on the type of milk they received: (1) only human milk: MOM and/or DHM), (2) mixed: MOM and/or DHM + FM, (3) only FM. We paid particular attention to children fed with donor human milk during hospitalization and separately assessed how they were fed at discharge.

### 2.10. Statistical Analysis

Initially, descriptive analyses were performed. To express categorical variables, we used the frequency and percentage (%). To express quantitative variables, the median and interquartile range (Q1–Q3) were used, as the data did not follow a normal distribution (data distribution checked with the Shapiro–Wilk test).

The Chi-square test or Fisher’s exact test were used to determine if two categorical variables were associated. Fisher’s exact test was used for small sample sizes. We used an alpha of 0.05. The differences were considered statistically significant when the *p*-value was <0.05. Statistica 13.3 Package (TIBCO Software Inc., San Ramon, CA, USA) was used to perform the statistical analysis.

## 3. Results

### 3.1. The Characteristics of the Study Group

Between 1 January 2022 and 31 March 2025 (excluding two first months of 2024), 527 neonates were admitted to the Neonatal Intensive and Intermediate Care Units of the Department of Neonatology.

In total, 499 neonates (94.69%) were subjected to further analysis, and their characteristics are presented in Table 1. The number of neonates analyzed in particular years was as follows: 2022-171, 2023-168, the first year of activity of the Human Milk Bank-160.

### 3.2. The Assessment of First Feeding

**Figure 1:**
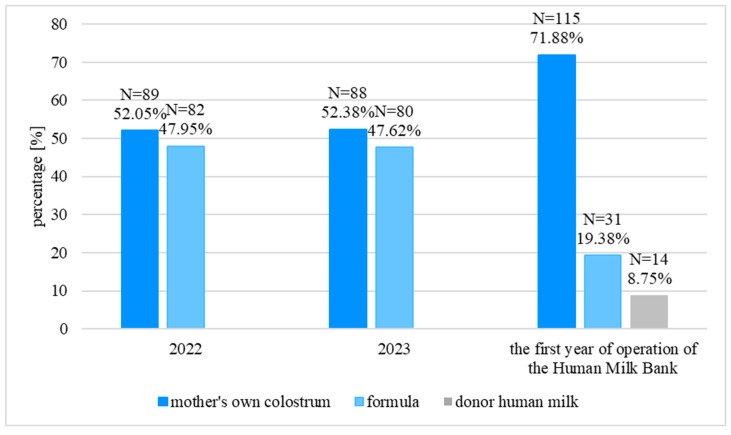
The type of first feeding.

There were no significant differences in the type of first feeding between 2022 and 2023 (*p* = 0.951, Chi-squared test). Similar percentages of neonates were fed MOC and FM (MOC vs. FM: 2022—52.05% vs. 47.95%; 2023—52.38% vs. 47.62%). In contrast, in the first year of operation of the HMB, the percentage of neonates fed MOC was significantly higher (71.88%; first year of operation of the HMB vs. 2023: *p* < 0.001; first year of the operation of the HMB vs. 2022: *p* < 0.001, Chi-squared test), and the percentage of neonates fed FM decreased (19.38%; the first year of operation of the HMB vs. 2023: *p* < 0.001; the first year of operation of the HMB vs. 2022: *p* < 0.001, Chi-squared test). Additionally, 8.75% of the neonates were fed DHM. The detailed data are presented in Figure 1.

### 3.3. The Assessment of Feeding During Hospitalization

The percentages of neonates fed MOM, MOM + FM and FM did not differ significantly between 2022 and 2023 (*p* = 0.802, *p* = 0.660, *p* = 0.730, respectively, Chi-squared test). The percentage of neonates fed MOM was significantly higher in the first year of operation of the HMB than in 2022 and 2023 (*p* = 0.004 and *p* < 0.001, respectively, Chi-squared test). Moreover, 12.50% (N = 20) of neonates were given MOM + DHM. When compared to 2022 and 2023, in the first year of operation of the HMB, a lower percentage of neonates were fed MOM + FM (*p* < 0.001 in both cases, Chi-squared test), the percentage of neonates fed FM did not differ significantly (*p* = 0.897 and *p* = 0.833, respectively, Chi-squared test). The detailed data are presented in Table 2.

We analyzed how the neonates who received DHM (N = 48) were fed during hospitalization. Here, 40.82% (N = 20) of them were fed MOM, 44.90% (N = 22) received MOM + FM, and 12.24% (N = 6) were fed FM.

**Figure 2:**
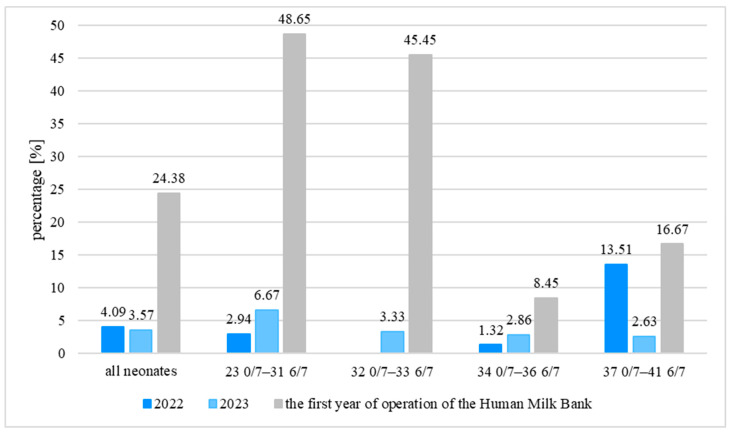
The type of feeding during hospitalization in individual gestational age groups.

The percentage of neonates fed HM was also analyzed in particular years taking into account the gestational age of the neonates. When compared to 2022, in the first year of operation of the Human Milk Bank, the percentage of neonates fed HM was significantly higher in the group of neonates aged 23 0/7–31 6/7 and 32 0/7–33 6/7 weeks of gestation (*p* < 0.001 in both cases, Fisher’s Exact Test). Among neonates aged 34 0/7–36 6/7 and 37 0/7–42 6/7 weeks of gestation, the percentage of neonates fed HM did not differ significantly (*p* = 0.115 and *p* = 0.743, respectively, Fisher’s Exact Test). The detailed data are presented in Figure 2.

**Figure 3:**
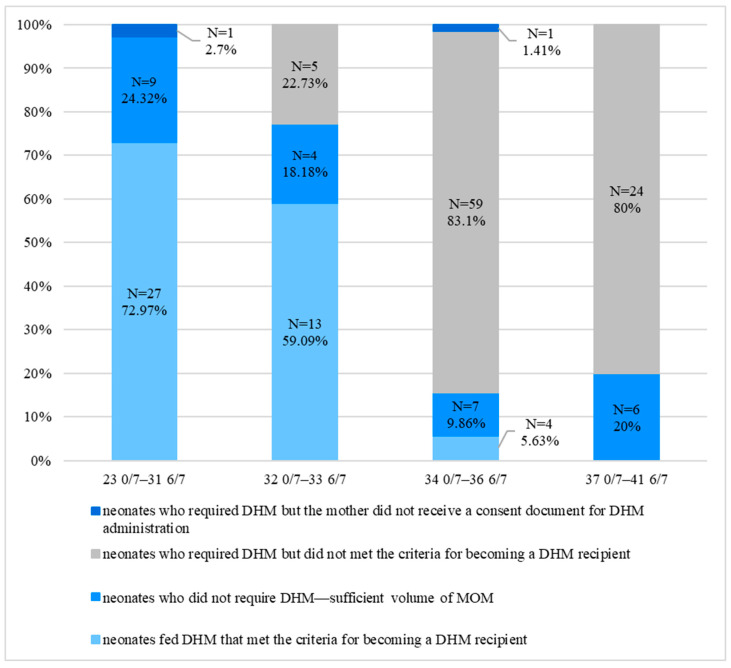
The type of feeding during hospitalization taking into account the criteria for becoming a donor human milk recipient. DHM—donor human milk, MOM—mother’s own milk.

Then, we wanted to check what percentage of neonates required/did not require DHM and what percentage of them finally received it. This was assessed in individual gestational age groups. The detailed data are presented in Figure 3. The percentage of neonates fed DHM who met the criteria for becoming a DHM recipient decreased with an increase in gestational age (23 0/7–31 6/7 vs. 34 0/7–36 6/7 *p* < 0.001, Fisher’s Exact Test). In contrast, the percentage of neonates who required DHM but did not meet the criteria for becoming a DHM recipient was higher in older neonates (32 0/7–33 6/7 vs. 37 0/7–42 6/7 *p* < 0.001, Fisher’s Exact Test).

### 3.4. The Assessment of Feeding at Discharge from the Department

**Figure 4:**
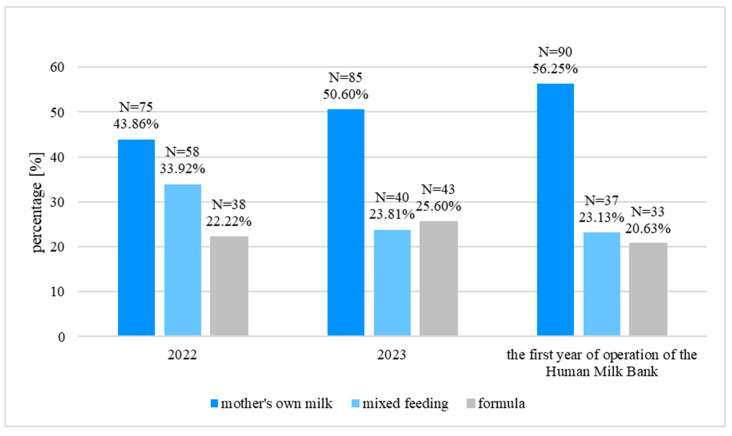
The type of feeding at discharge from the Department.

The percentage of neonates fed MOM at discharge increased significantly between 2022 and the first year of operation of the HMB (43.86% vs. 56.25%, *p* = 0.024, Chi-squared test). The percentage of neonates fed FM did not differ between the periods studied (*p* > 0.05 in each case, Chi squared test). The percentage of neonates fed both MOM and FM decreased significantly between 2022 and 2023 (33.92% vs. 23.81%, *p* = 0.040, Chi-squared test) and remained similar to 2023 in the first year of operation of the HMB (23.13% vs. 23.81%, *p* = 0.884, Chi-squared test). The detailed data are presented in Figure 4.

We also analyzed how the neonates who received DHM (N = 48) were fed at discharge from the Department. Here, 53.06% (N = 26) of them were fed MOM, 22.45% (N = 11) were fed FM, and 22.45% (N = 11) received both MOM and FM.

### 3.5. Summary of Findings

In the first year of operation of the HMB, the percentage of neonates who received MOC was significantly higher (71.88% vs. 52.28% (2023) and 52.05% (2022); *p* < 0.001 in both cases, Chi-squared test). In addition, the percentage of neonates fed MOM during hospitalization was significantly higher (11.88% vs. 3.57% (2023) and 4.09% (2022); *p* = 0.004 and *p* = 0.009, respectively, Chi-squared test). Moreover, 12.50% of neonates were given MOM + DHM. The percentage of neonates fed MOM at discharge increased significantly between 2022 and the first year of operation of the HMB (43.86% vs. 56.25%, *p* = 0.024, Chi-squared test). Here, 53.06% of neonates who received DHM were fed MOM at discharge.

## 4. Discussion

The establishment of the Human Milk Bank of the University Clinical Hospital of Bialystok in 2024 has significantly modified feeding practices in the Neonatal Intensive and Intermediate Care Units of the Department of Neonatology. The possibility of using DHM did not limit the desire to feed neonates with MOM but significantly intensified it.

In the first year of operation of the HMB, significantly higher percentages of neonates received MOC (71.88% vs. 52.28% in 2023 and 52.05% in 2022). It is especially important when compared to the fourth quarter of 2021, when only 12% of neonates received MOC in the first feeding. Moreover, in the first year of operation of the HMB, a significantly higher percentage of neonates were fed MOM during hospitalization (11.88% vs. 3.57% in 2023 and 4.09% in 2022) and at discharge from the Department (56.25% vs. 50.06% in 2023 and 43.86% in 2022). Our findings are consistent with previous studies, which showed an elevated rate of exclusive breastfeeding during hospitalization after the introduction of HMB [12,13,14,15]. A Polish study by Lehman et al. showed that DHM supply during the first year of a HMB operation did not influence lactation or breastfeeding negatively [16]. These results show that the introduction of DHM feeding practice does not lead to a reduction in MOM administration. In contrast, it may elevate the proportion of neonates fed exclusively MOM. As the idea of human milk banking has become popular worldwide, the question of whether the use of DHM and the presence of HMB can threaten both mother’s and staff commitment to promote breastfeeding [15]. Our results, as well as the available evidence contradicts the hypothesis about the adverse effects of DHM on the stimulation of lactation and breastfeeding rates [17].

A study by Wilunda et al. showed that integrating lactation support and human milk banking may improve the exclusive use of MOM [18]. Therefore, HMBs are widely recommended to improve the availability of not only DHM but especially MOM [19]. They should be seen as a tool for promoting the use of human milk, breastfeeding and lactation [12]. Medical staff, observing the difficulties of milk banking processes, from the recruitment of donors to the freezing of pasteurized milk, feel more deeply the importance of feeding neonates human milk. This translates into supporting mothers to feed their children with their own milk [13]. Moreover, mothers, who asked for their consent to feed their neonate DHM if their milk supply is low, are always educated about the entire procedure and may feel more connected to the importance of feeding their child human milk. This translates into stimulating their own lactation [13]. We considered the influence of other factors on the demonstrated improvement in the quality of neonatal nutrition. However, we did not observe any significant changes in feeding practices within the Department. There was no increase in the number of employed doctors, midwives or lactation consultants, no modifications were made to the “Early Lactation Stimulation Program”, and no new detailed recommendations were implemented.

Our study showed that as many as 53.06% of neonates who received DHM during hospitalization were fed exclusively MOM at discharge. Helping mothers maintain human milk provision through discharge is crucial [15]. In addition to active education, the availability of DHM may play an important role in influencing maternal choices and may lead to increased MOM feeding at discharge [13]. This can motivate the mother to improve her pumping technique until the neonate is able to breastfeed [15]. However, despite a clear improvement in feeding human milk at discharge, challenges remain in sustaining this success after discharge. This should become the subject of discussion aimed at implementing effective educational programs [20].

In our study, the percentage of neonates fed exclusively HM (MOM + DHM) during hospitalization increased significantly after the introduction of HMB but still reached only 24.38%. This is due to the differences in gestational age of the neonates studied. Here, 43.49% of the children were late preterm neonates (34 0/7–36 6/7 weeks of gestation). Unlike younger premature neonates, they need much larger volumes of milk for effective early enteral nutrition, but their mothers often cannot obtain a sufficient amount of breast milk [15]. In our study, only 9.86% of mothers achieved this. According to the criteria for becoming a DHM recipient, only 5.63% of neonates at this gestational age received DHM. In the study by F. Mongelli et al., 3 years after the introduction of a HMB, the exclusive HM feeding of neonates with birth weight <1500 g increased significantly, reaching 47.2% in 2024 [13]. In our study, a significant increase in HM feeding was observed not only in the group of the youngest neonates aged 23 0/7–31 6/7 weeks of gestation (48.65%) but also in the group of older neonates aged 32 0/7–33 6/7 weeks of gestation (45.45%).

During our study, we noticed that there is a group of children who need DHM but do not receive it. This can happen when a mother does not receive a consent document for DHM administration, or a neonate does not meet the criteria for becoming a DHM recipient. In the case of the first group of children, every effort should be made to ensure that each mother receives the appropriate documents. In the case of the second group, efforts should be made to expand very restrictive criteria of the government program ‘’For Life” used in Poland, under which the hospital receives reimbursement of the costs of feeding neonates DHM, according to the following: (1) gestational age of 23 0/7–36 6/7 weeks, (2) chronological age of ≤28 days, (3) feeding via gastric tube (all feedings within 24 h) [11]. In particular, the requirement to meet the third point (feeding via gastric tube (all feedings within 24 h)) urgently needs to be revised. It prevents late preterm infants from benefiting from DHM if they cannot be fed MOM [13]. With this in mind, we should strive to expand the list of potential DHM recipients to reach as many newborns as possible.

The American (American Academy of Pediatrics), Italian (Italian Association of Human Milk Banks) and English (East of England Neonatal Operational Delivery Network) recommendations regarding DHM recipients are usually ranked according to the neonate’s clinical condition, with priority given to neonates born <32 weeks of gestation and weighing <1500 g. However, if the HMB has an adequate volume of milk, it should be administered to subsequent groups of recipients. One of these groups includes neonates born >32 weeks of gestation who need a ‘’bridge’’ milk supply, if their mother has a clear intention to start breastfeeding [21,22,23]. This is a very valuable recommendation that significantly expands the group of neonates who can benefit from DHM.

Assessing the impact of milk banking on feeding practices requires further research. Firstly, in the context of the HMB of the University Clinical Hospital of Bialystok, a detailed analysis of the subsequent years of operation as well as predictive analyses seem necessary to assess its operation and effectiveness. Secondly, it would be important to determine whether newborns who were fed MOM and DHM during hospitalization achieved exclusive MOM feeding earlier compared to those who were fed MOM and FM. This would allow us to determine whether DHM administration contributes to earlier exclusive breastfeeding. Thirdly, in the context of HMBs in general, multicenter prospective studies should be conducted in different parts of the world to thoroughly understand the role of human milk banking in shaping feeding practices in Departments of Neonatology.

## 5. Strengths and Limitations

We assessed the impact of the newly opened Human Milk Bank on feeding practices in the tertiary referral Neonatal Intensive and Intermediate Care Unit. It is significant that this is the first HMB in northeastern Poland. Thanks to this analysis, we can understand the impact of this type of facility on feeding practices among mothers who have limited knowledge about HMB. It is also the first study of this type covering the mentioned region of the country.

This study has several limitations. This is a retrospective study, which does not allow conclusions regarding causality; we can only establish associations. In addition, a time trend could be a possible confounding factor. However, during the study period, we did not notice any significant changes in the functioning of the Department (in terms of breastfeeding promotion), and there were no widespread breastfeeding promotion campaigns in Poland which could change attitudes towards breastfeeding. The period following the opening of the HMB and the sample size was small. Therefore, we cannot accurately assess long-term outcomes and trends. Our study is based on retrospective data; therefore, potential errors related to data collection cannot be ruled out despite all efforts. It was a single-center study; therefore, its results should not be generalized. Culture deeply influences feeding practices and determines attitudes towards breastfeeding and human milk banking. The trends we assess may also be influenced by the characteristics of the research center and the HMB. Additionally, we only analyzed data that extended to the day of discharge from the Department. Therefore, we are unable to assess post-discharge breastfeeding and long-term effects, which are extremely important, because maintaining breastfeeding after discharge should be a priority.

## 6. Conclusions

The possibility of using milk from the HMB did not limit the desire to feed neonates MOM but intensified it. Neonates were more likely to be fed MOM during the first feeding, throughout their hospitalization, and at discharge. A high percentage of children who received milk from the HMB were fed exclusively MOM at discharge. Our data show the strong potential of HMBs in improving feeding practices in Intensive and Intermediate Care Units. Additionally, it indicates the need to expand the criteria for becoming a DHM recipient, so that every neonate in need has the opportunity to benefit from DHM.

## Figures and Tables

**Table 1:** The characteristics of the study group.

Characteristic	All Neonates (*n* = 499)
	Frequency (Percentage)
term vs. preterm birth	105 (1.04%) vs. 394 (78.96%)
23 0/7–31 6/7 WG	101 (20.24%)
32 0/7–33 6/7 WG	76 (15.23%)
34 0/7–36 6/7 WG	217 (43.49%)
birth weight < 2500 g vs. ≥2500 g	314 (62.93%) vs. 185 (37.07%)
<1000 g	23 (4.61%)
1000–1499 g	68 (13.63%)
1500–1999 g	113 (22.65%)
2000–2499 g	109 (21.84%)
vaginal delivery vs. cesarean section	148 (29.66%) vs. 351 (70.34%)
inborn vs. outborn	468 (93.79%) vs. 31 (6.21%)
single vs. multiple pregnancy	367 (73.55%) vs. 132 (26.45%)
primigravida vs. multigravida	187 (37.47%) vs. 312 (62.53%)
primiparous vs. multiparous	226 (45.29%) vs. 273 (54.71%)
	Median (Q1–Q3)
gestational age (weeks)	35 (32–37)
birth weight (g)	2160.00 (1720.00–2770.00)

Q1—first quartile, Q3—third quartile, WG—weeks of gestation.

**Table 2:** The type of feeding during hospitalization.

Type of Feeding	2022 (N = 171)	2023 (N = 168)	The First Year of Operation of the HMB (N = 160)	p1	p2	p3
MOM	7 (4.09%)	6 (3.57%)	19 (11.88%)	0.802	0.004	0.009
MOM + DHM	NA	NA	20 (12.50%)	NA	NA	NA
MOM + FM	149 (87.13%)	149 (88.69%)	80 (50.00%)	0.660	<0.001	<0.001
MOM + DHM + FM	NA	NA	22 (13.75%)	NA	NA	NA
DHM + FM	NA	NA	6 (3.75%)	NA	NA	NA
FM	15 (8.77%)	13 (7.74%)	13 (8.13%)	0.730	0.897	0.833

HMB—Human Milk Bank; MOM—mother’s own milk; DHM—donor human milk; FM—formula; NA—non-applicable; p1—comparison of year 2022 and 2023; p2—comparison of year 2023 and the first year of operation of the HMB; p3—comparison of year 2022 and the first year of operation of the HMB.

## Data Availability

The data that support the findings of this study are not openly available due to reasons of sensitivity and are available from the corresponding author upon reasonable request.
